# Crinivirus replication and host interactions

**DOI:** 10.3389/fmicb.2013.00099

**Published:** 2013-05-20

**Authors:** Zsofia A. Kiss, Vicente Medina, Bryce W. Falk

**Affiliations:** ^1^Department of Plant Pathology, University of CaliforniaDavis, CA, USA; ^2^Department of Crop and Forest Sciences, University of LleidaLleida, Spain

**Keywords:** phloem-limited, plasmalemma deposit, whitefly vector, *Crinivirus*, quintuple gene block

## Abstract

Criniviruses comprise one of the genera within the family *Closteroviridae*. Members in this family are restricted to the phloem and rely on whitefly vectors of the genera *Bemisia* and/or *Trialeurodes *for plant-to-plant transmission. All criniviruses have bipartite, positive-sense single-stranded RNA genomes, although there is an unconfirmed report of one having a tripartite genome. *Lettuce infectious yellows virus* (LIYV) is the type species of the genus, the best studied so far of the criniviruses and the first for which a reverse genetics system was developed. LIYV RNA 1 encodes for proteins predicted to be involved in replication, and alone is competent for replication in protoplasts. Replication results in accumulation of cytoplasmic vesiculated membranous structures which are characteristic of most studied members of the *Closteroviridae*. These membranous structures, often referred to as *Beet yellows virus* (BYV)-type vesicles, are likely sites of RNA replication. LIYV RNA 2 is replicated *in trans* when co-infecting cells with RNA 1, but is temporally delayed relative to RNA 1. Efficient RNA 2 replication also is dependent on the RNA 1-encoded RNA-binding protein, P34. No LIYV RNA 2-encoded proteins have been shown to affect RNA replication, but at least four, CP (major coat protein), CPm (minor coat protein), Hsp70h, and P59 are virion structural components and CPm is a determinant of whitefly transmissibility. Roles of other LIYV RNA 2-encoded proteins are largely as yet unknown, but P26 is a non-virion protein that accumulates in cells as characteristic plasmalemma deposits which in plants are localized within phloem parenchyma and companion cells over plasmodesmata connections to sieve elements. The two remaining crinivirus-conserved RNA 2-encoded proteins are P5 and P9. P5 is 39 amino acid protein and is encoded at the 5′ end of RNA 2 as ORF 1 and is part of the hallmark closterovirus gene array. The orthologous gene in BYV has been shown to play a role in cell-to-cell movement and indicated to be localized to the endoplasmic reticulum as a Type III integral membrane protein. The other small protein, P9, is encoded by ORF 4 overlaps with ORF 3 that encodes the structural protein, P59. P9 seems to be unique to viruses in the genus *Crinivirus*, as no similar protein has been detected in viruses of the other two genera of the *Closteroviridae*.

## INTRODUCTION

Most plant viruses have positive-sense single-stranded RNA (ssRNA) genomes that vary in size among viruses in different taxa. Members in the family *Closteroviridae* possess the largest and most complex ssRNA genomes which vary in size from ca. 15–20 kb (Martelli et al., 2012a). Closteroviruses (the generic name for viruses in the family) are currently placed within three approved and one proposed genera (Martelli et al., 2012a). The genus *Closterovirus* contains viruses whose genomes are monopartite, and that are transmitted to plants by various aphid vectors. The genus *Crinivirus* encompasses viruses whose genomes are bipartite (although one member has a proposed tripartite genome). Criniviruses are exclusively transmitted by whiteflies of two genera: *Bemisia* and *Trialeurodes*. The genus *Ampelovirus* has members with monopartite genomes, and the viruses are transmitted by mealybugs. The newly proposed genus, *Velarivirus*, contains members formerly within the genus *Ampelovirus*, but which represent a different phylogenetic clade (Martelli et al., 2012b). However, despite these genomic and biological differences all closteroviruses possess many commonalities. All members have characteristic long, flexuous rod-shaped virions, which range in size from ca. 750–2000 nm, depending on the specific virus. All closteroviruses share two conserved gene modules including one encoding proteins associated with replication (ORFs 1A and 1B), and the quintuple gene block, or the “hallmark closterovirus gene array” encoding for proteins that are not associated with replication, but are virion components or are involved in other biological processes of closterovirus infections. For criniviruses, these two gene modules are separated onto the two distinct genomic RNAs, and at least for one crinivirus the separation of these gene modules likely plays a role in temporal regulation of genome replication and gene expression.

**FIGURE 1:**
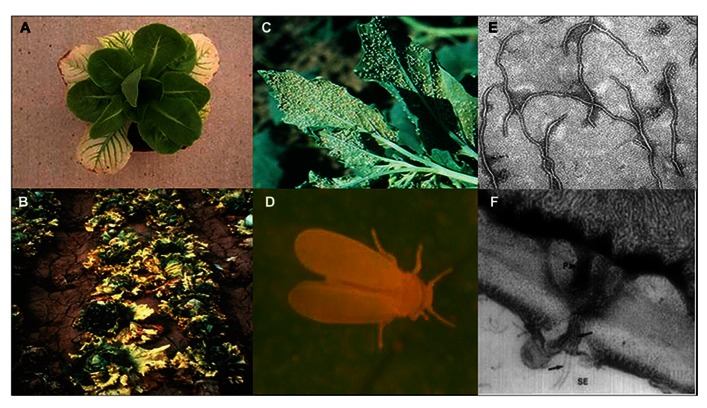
***Lettuce infectious yellows virus* (LIYV) infected lettuce plants close up (A) and field shot (B)**. The sweetpotato whitefly, *Bemisia tabaci* New World (formerly called biotype A) colonizing a *Chenopodium* spp. plant in the field **(C)** and close up **(D)**. LIYV virions by transmission electron microscopy **(E)** and **(F)** a thin section showing cross section of pore-plasmodesma connecting sieve element and phloem parenchyma cell, showing flexuous rod virions (black arrows) within plasmodesma and in both cells, and plamalemma deposits on the phloem parenchyma cell membrane above plasmodesmatal pore (adapted from Hoefert et al., 1988 with permission from Elsevier).

*Lettuce infectious yellows virus* (LIYV) is the type member of the genus *Crinivirus*. Studies on LIYV date back to the late 1970s when several crops in California and Arizona [including lettuce (*Lactuca sativa*; **Figure 1A**), melons (*Cucumis melo*), and sugar beets (*Beta vulgaris*)], were severely affected by this newly discovered virus, resulting in losses exceeding $20 million in a single growing season (Flock and Duffus, 1982). Due to the severe economic losses caused by LIYV at that time, LIYV became a subject of intense investigations. By 1982, it was recognized as a distinct and emerging “new” virus and was found to be associated with the rapid expansion and spread of the sweet potato whitefly, *Bemisia tabaci* biotype A (now New World; Flock and Duffus, 1982; De Barro et al., 2011; **Figure 1D**). Primary work focused on characterizing LIYV whitefly transmission properties, host range, examination of virion morphology, and its effect on host cells (Duffus et al., 1986; Hoefert et al., 1988). Advances in DNA sequencing and molecular biology demonstrated the bipartite nature of the LIYV genome. LIYV was the first crinivirus whose genome was sequenced and was the first for which reverse genetics approaches were developed that further enabled studies of replication, gene expression, and protein functions (Klaassen et al., 1995, 1996). Although today LIYV is not agriculturally important due in part to displacement of the *Bemisia tabaci* biotype A (New World) by a more competitive, and more aggressive non-LIYV vector whitefly, *Bemisia tabaci* biotype B (now called Middle East/Asia Minor; De Barro et al., 2011), studies on LIYV continued and have proved to be critical in establishing a basic understanding of crinivirus–host and crinivirus–vector interactions. These efforts also aided further studies with other criniviruses, many of which are currently of great economic importance. Here, we intend to review these seminal studies that allowed the development of current understanding of LIYV/crinivirus replication and host plant interactions.

## LIYV AS THE SEMINAL CRINIVIRUS

*Lettuce infectious yellows virus* was discovered coincident with the explosion of the *Bemisia tabaci *biotype A (New World) population in southern California and Arizona in the late 1970s. Although whiteflies, and particularly, *Bemisia tabaci* had been recognized as a plant virus vector for many years, LIYV was recognized as a novel type of virus at that time. *Bemisia tabaci*-mediated LIYV transmission was semi-persistent. Transmission electron microscopic studies on purified virions and LIYV-infected plants showed that LIYV virus like particles (virions) were similar to those of the closteroviruses known at that time. The virions were long, flexuous rods (**Figure 1C**; Duffus et al., 1986) and in plants the virions and cytopathologies of infection were limited to phloem cells (Hoefert et al., 1988). Although initial virion size estimates suggested lengths of ~2000 nm for LIYV (Duffus et al., 1986) similar to lengths of known aphid-transmitted closteroviruses including *Beet yellows virus* (BYV) and *Citrus tristeza virus* (CTV), subsequent studies revealed that LIYV has shorter particle lengths of approximately 800 nm (Tian et al., 1999), and further studies on other later-discovered criniviruses showed similar virion lengths (Liu et al., 2000). We now know that these lengths reflect the sizes of the encapsidated genomic RNAs.

Virion purification and RNA extraction and analysis showed another unique feature; purified LIYV virion preparations contained two distinct ssRNA molecules of 8,118 nucleotides and 7,193 nucleotides, respectively, thus, suggesting that LIYV has a bipartite genome (Klaassen et al., 1994). This was in contrast to the other closteroviruses that were characterized at that time [e.g., BYV, CTV, and *Beet yellow stunt virus* (BYSV)] all of which had a single large, single-stranded genomic RNA (Bar-Joseph and Hull, 1974; Dodds and Bar Joseph, 1983; Reed and Falk, 1989). By 1995, both of the LIYV genomic RNAs were sequenced (Klaassen et al., 1995), which enabled comparisons of the LIYV genomes with those of BYV and CTV, the only other closteroviruses sequenced at that time. Comparison of deduced protein amino acid sequences with those of other filamentous plant viruses showed that the LIYV major coat protein (CP) sequence was most similar to the coat protein sequences of BYV and CTV (Klaassen et al., 1994) and allowed for a more precise taxonomic classification of LIYV, which led to the establishment of the genus *Crinivirus* within the family *Closteroviridae*. The genus name *Crinivirus*, comes from the latin “crinis” for “hair” (Martelli et al., 2002).

**FIGURE 2:**
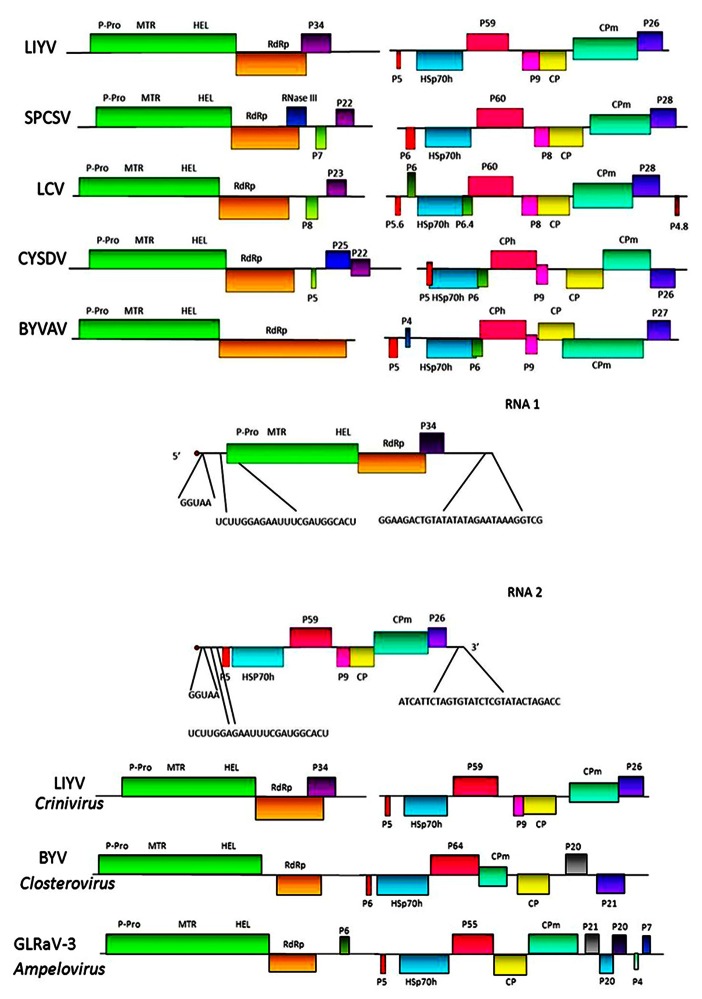
**Upper section shows genome maps for the bipartite genomic RNAs for five criniviruses**. LIYV = *Lettuce infectious yellows virus*; SPCSV = *Sweet potato chlorotic stunt virus*; LCV = *Lettuce chlorosis virus*; CYSDV = *Cucurbit yellow stunting disorder virus*, and BYVAV = *Blackberry yellow vein associated virus*. Colored boxes indicate specific ORFs. P-Pro – papain-like protease; MTR = methyl-transferase; HEL = helicase; RdRp = RNA-dependent RNA polymerase; HSp70h = heat shock protein 70 homolog; CP and CPm = major and minor capsid proteins, respectively. Other ORFs labeled with P and a number indicate proteins and their approximate molecular mass (P26 = a 26 kDa protein). Middle section shows the comparative nucleotide sequences at the 5′ and 3′ terminal regions of the LIYV genomic RNAs 1 and 2. Lower section shows for comparison genomic maps of viruses in the two other genera of the family *Closteroviridae*: *Closterovirus* and *Ampelovirus*.

Sequencing the LIYV genomic RNAs showed that the LIYV RNAs 1 and 2 contained the gene modules that are characteristic of BYV and CTV, but also showed them to be separated between the two genomic RNAs, 1 and 2 (**Figure 2**). Later sequencing of other crinivirus genomes showed that they also have bipartite genomes with conservation of most of the gene content and order (**Figure 2**) with the possible exception of *Potato yellow vein virus* (PYVV), which is suggested to have a tripartite genome (Livieratos et al., 2004).

## GENOME ORGANIZATION

*Lettuce infectious yellows virus* RNA 1 is 8,118 nt and contains a 5′ cap structure and the 3′ terminus is not polyadenylated. RNA 1 includes a 97 nucleotide 5′ untranslated region followed by two ORFs, 1A and 1B. ORF 1A encodes a potential protein of 1873 amino acids. Alignment of the ORF 1A protein amino acid sequences of LIYV with those of the BYV ORF 1A protein showed that the highest sequence similarity was in the methyl-transferase (MTR) and RNA helicase (HEL) motifs (Klaassen et al., 1995). Although amino acid sequences upstream of the MTR domain did not show statistically significant similarity, there were motifs identified for both LIYV and BYV that showed the characteristic signature of papain-like proteases (Agranovsky et al., 1994; Klaassen et al., 1995; Peremyslov et al., 1998). Agranovsky et al. (1994) performed site directed mutagenesis of this region for BYV and showed that the catalytic cysteine residue of the leader protease is contained in this motif. It is suggested that the homologous cysteine residue of LIYV has the same role (Peng et al., 2001). Further studies showed that the leader proteinase of BYV is important for genome amplification and required for long-distance transport of the virus within plants (Peng and Dolja, 2000; Peng et al., 2003). ORF 1B encodes a putative protein that shows characteristic motifs of a RNA-dependent RNA polymerase (RdRp). It is believed that the RdRp domains of BYV and CTV are expressed directly from the genomic RNA itself via a +1 ribosomal frameshift event (Agranovsky et al., 1994; Dolja et al., 1994). Genome structure analysis, and alignment of the amino acid sequences of LIYV and BYV around the overlap region of ORFs 1A and 1B showed that the C-terminal portion of BYV ORF 1A aligned with the N-terminal portion of LIYV ORF 1B, and thus the potential frameshift sites in these two viruses are not homologous. Translation frameshift events are found for several plant viruses, but these are typically a -1 frameshift and these have been well studied (Dreher and Miller, 2006). However, mechanisms have been described for +1 frameshifting in retrotransposons and some other viruses. These appear to be much simpler than those for a -1 frameshift and need not be associated with distinct structural features (Farabaugh et al., 1993). Studies have indicated that in viruses that are in the family *Closteroviridae* this +1 frameshifting may occur at a conserved GUU_stop_C motif at the ORF 1 stop codon and this +1 slippage is likely to occur at the P-site from GUU to UUU with a stop codon in the A-site. However, even to this there is exception as in the case of CTV where the frameshifting occurs upstream of the ORF 1 stop codon and it has been precisely shown that the frameshifting occurs at the GUU_CGG_C sequence which aligns with the GUU_stop_C motif in other closteroviruses (Firth and Brierley, 2012). Other criniviruses show very similar organization for ORF 1A and 1B. Downstream of ORF 1B, LIYV RNA 1 contains ORF 2. The protein encoded by this ORF, P34, shows no similarity to any proteins in the National Center for Biotechnology Information (NCBI) database (www.ncbi.nlm.nih.gov), but shows analogy in respect to its size and location to ORF 2 of CTV and BYSV (Dolja et al., 1994; Karasev et al., 1994) that belong to the genus *Closterovirus*. However, other criniviruses also encode proteins on RNA 1 downstream of ORF 1B, but these vary in size and possible function (**Figure 2**).

For example, *Sweet potato chlorotic stunt virus* (SPCSV) has been shown to encode a protein, P22, from the 3′ terminal ORF on RNA 1. P22 exhibits RNase III endonuclease activity and *in vitro* has been shown to cleave double-stranded small interfering RNAs (Cuellar et al., 2009). In plants P22 functions as an RNA silencing suppressor protein, even suppressing resistance against the unrelated potyvirus, *Sweet potato feathery mottle virus *(Cuellar et al., 2009). Interestingly, not all isolates of SPCSV contain an ORF encoding for P22 (Cuellar et al., 2008). Multiple suppressors of RNA silencing have also been shown to be encoded by *Tomato chlorosis virus* (ToCV). One of these, P22, is encoded by the ToCV RNA 1 3′ ORF, similarly to that seen for SPCSV (Canizares et al., 2008). And although the 3′ end of RNA 1 does not show nucleotide sequence homology to other criniviruses the fact that other 3′ end proteins in other criniviruses show silencing suppressor activity motivated us to test if P34 could be a potential silencing suppressor. However, so far we have no evidence indicating in 16c *Nicotiana benthamiana* assays suggesting that P34 could be a potential silencing suppressor.

**FIGURE 3:**
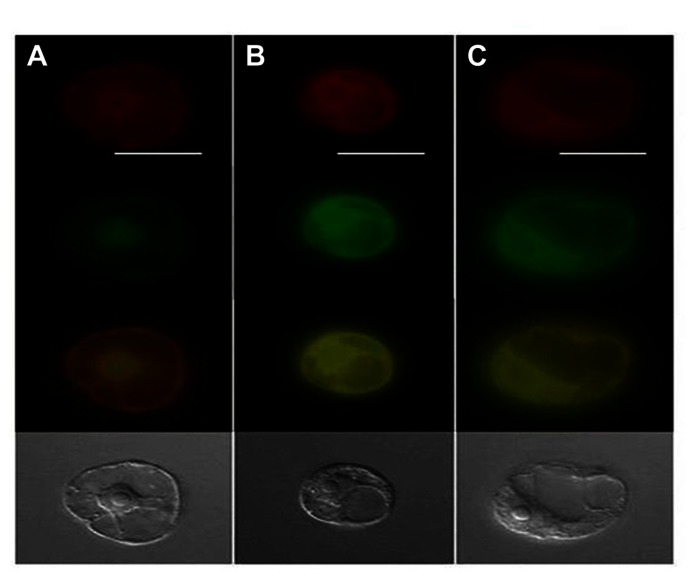
**Epifluorescence microscopy of endoplasmic reticulum (ER) and GFP localization where ER (red) on top panel, GFP (green) in second panel, merged green/red in third panel and the bottom panel displays the transmitted light images**. Panel **(A)** shows TMV 30B-GFPc3 (GFPc3 is GFP where ER localization signal have been removed) inoculated cells while **(B)** shows cells that were inoculated with TMV GFP:P34, and **(C)** is cells inoculated with TMV P34:GFP. Image from Wang et al. (2010) with permission.

*Lettuce infectious yellows virus* P34 is likely to be translated from a highly abundant subgenomic RNA, which is the most abundant LIYV-specific RNA found within LIYV-infected cells (Yeh et al., 2000). Subsequent studies have shown that P34 is an RNA-binding protein and plays an important role in the replication of LIYV RNA 2 (Yeh et al., 2000; Wang et al., 2010). Further evidence suggesting that P34 might play a role in LIYV RNA replication was shown by its localization to the endoplasmic reticulum (ER) and to the perinuclear envelope (Wang et al., 2010; **Figure 3**).

*Lettuce infectious yellows virus* RNA 2 is 7,193 nt, 5′ capped, not polyadenylated and does not encode proteins necessary for RNA replication. RNA 2 contains a 5′untranslated sequence of 326 nt. This sequence only shows limited homology with LIYV RNA 1 including the first five nucleotides (5′-GGUAA-3′) and a stretch of 23 nucleotides (5′-UCUUGGAGAAUUUCGAUGGCACU-3′). These 23 nucleotides in RNA 1 are from positions 83 to 105 and surround the first AUG which begins at nucleotide 99. However, in LIYV RNA 2 this 23 nt stretch is found upstream (at positions 121–143) of the first AUG start codon that is located at position 327(Klaassen et al., 1995; **Figure 2**). The significance of the common 23 nucleotide sequence is not known. It is important and worthwhile to note here, that LIYV RNA 1 and RNA 2 show no nucleotide sequence homology in their 3’-termini, unlike that found for most multipartite plant viruses and most of the other criniviruses sequenced so far. ORF 1 of LIYV RNA 2 encodes for a small protein of ~5 kDa. This is the first ORF of the conserved closterovirus quintuple gene block, and the putative LIYV protein, as well as those for other closteroviruses is predicted to be highly hydrophobic and to form a transmembrane helix. ORF 2 encodes the Hsp70h. This is conserved among closteroviruses, and before many closterovirus genomes were sequenced, conservation among the specific motifs shared among heat shock 70 proteins was used to design degenerate oligonucleotide primers that proved to be very useful for generic closterovirus detection (Karasev et al., 1994; Tian et al., 1996). Interestingly, additional conservation between the Hsp70h-related proteins of closteroviruses was observed in the C-terminal regions indicating that these domains might be involved in protein–protein interactions, and may be important for chaperone activity (Klaassen et al., 1995). LIYV RNA 2 ORF 3 encodes a protein, P59, that shows significant similarities with the deduced amino acid sequences of CTV P61 (Pappu et al., 1994) and BYV P64 (Agranovsky et al., 1991; Klaassen et al., 1995). ORF 4 overlaps with ORF 3 and encodes a small protein, P9. LIYV ORF 4 is not part of the closterovirus quintuple gene block, but a similarly positioned ORF encoding for a similarly sized protein is conserved among criniviruses (Dolja et al., 2006; **Figure 2**), but no function has yet been determined for this protein. Amino acid alignment of crinivirus P9 homologs does not show significant amino acid similarity, but the predicted secondary structures of these proteins are very similar (Stewart et al., 2009a,b). ORF 5 encodes the ~28 kDa LIYV coat protein (CP) which shows high similarity in sequence with coat protein sequences of BYV and CTV. The final quintuple gene block ORF, ORF 6 overlaps with ORF 5 and encodes a ~52 kDa protein, the CPm (minor coat protein) that is predicted to be a diverged, duplicated copy of the CP. The C-terminal half of this protein contains the R, G, and D amino acid residues shown to be invariant in all filamentous virus coat proteins (Dolja et al., 1991). It is interesting to note that the order of the CP and CPm ORFs are the same for all criniviruses, but opposite to the order of the respective ORFs found for viruses in the genus *Closterovirus*. Furthermore, while the CPs are similar in size among most closteroviruses, the respective sizes of the CPm proteins differ among the viruses in some of the genera. For example, for viruses in the genus *Closterovirus* the CPm is ca. 24 kDa while in the genus *Crinivirus *the CPm is much larger, ranging in size from ca. 53 kDa for LIYV ro ca. 77 kDa for *Dioda vein chlorosis virus *(Tzanetakis et al., 2011). Within the genus *Ampelovirus* and proposed genus *Velarivirus*, the CPms vary in size (and possibly numbers) for various members. LIYV RNA 2 ORF 7 encodes a 26 kDa protein, P26. Similarly positioned ORFs encoding similarly sized proteins are found among all criniviruses, and while, like for P9, the amino acid sequences do not show significant similarity, their predicted secondary structures are similar (Stewart et al., 2009a). LIYV RNA 2 most likely serves as an mRNA only for P5. Subgenomic RNAs for LIYV RNA 2 ORFs 2–7 have been identified from infected plants and protoplasts (Rubio et al., 2002). The genomic RNA 2 components for other criniviruses are similar in overall organization to that of LIYV (**Figure 2**).

## LIYV VIRIONS

**FIGURE 4:**
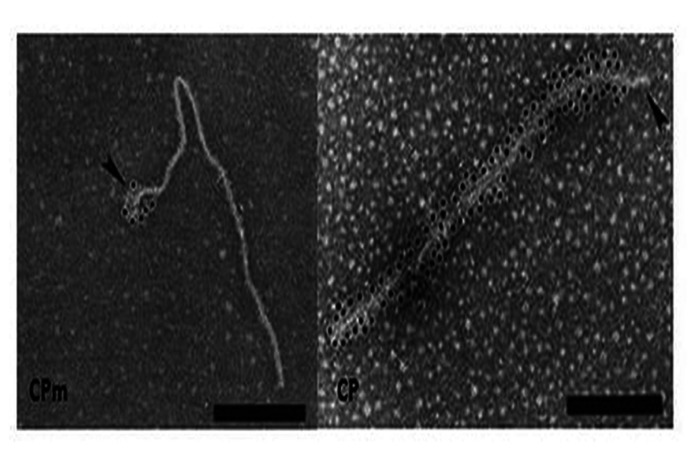
**Immunogold labeling of LIYV virions using antibody against CP and CPm**. Gold labeling is indicated by the black dots, note that the CPm only encapsidates a short segment at one end of the virion while CP composes the remainder of the capsid. Image from Tian et al. (1999) with permission.

Gaining the LIYV genome sequence information was a very important step for showing relationships of LIYV to other closteroviruses, and allowed for predicting potential roles of some LIYV-encoded proteins in LIYV infections. First, transmission electron microscopy (TEM) and immunogold labeling analyses confirmed that the LIYV virions, like those of BYV and CTV are morphologically polar (**Figure 4**). The CPm is localized to a short terminal region while the CP makes up the majority of the capsid. However, a surprising result was finding that the Hsp70h and P59 also are LIYV virion components suggesting even further complexity to LIYV and other closterovirus virions. This was first demonstrated when stringently purified LIYV virions were analyzed by SDS-PAGE and immunoblotting using antisera specific to four LIYV-encoded proteins: CP, CPm, P59, and to Hsp70h (Tian et al., 1999). However, TEM and immunogold labeling failed to allow for localizing the positions of P59 and Hsp70h on the virions (Tian et al., 1999). Later, elegant work with CTV and BYV suggested that the respective orthologous proteins form an interface on the capsid between the CP and CPm proteins (Peremyslov et al., 2004; Satyanarayana et al., 2004) This has not yet been demonstrated for any crinivirus.

Purified LIYV virions proved to be transmissible to plants by the whitefly, *Bemisia tabaci* biotype A (New World). This suggested that perhaps one or more of the four virion proteins might be a determinant of *Bemisia tabaci* transmissibility. Tian et al. (1999) used antisera to the four LIYV virion proteins to assess if they could interfere with, or neutralize, *in vitro* acquisition and subsequent transmission of LIYV by *Bemisia tabaci* biotype A to plants. Results from several experiments indicated that LIYV transmission efficiency was not affected by antisera to P59, CP, or Hsp70h, however, the CPm antiserum specifically and completely eliminated LIYV transmission by *Bemisia tabaci*. Only, when the CPm antiserum was diluted to 1% final concentrations was there incomplete neutralization of LIYV transmission by *Bemisia tabaci* biotype A (New World; Tian et al., 1999). This strongly suggested that the CPm had a primary role in LIYV transmissibility by *Bemisia tabaci *biotype A (New World). This has been supported by subsequent studies of Stewart et al. (2010), where they showed that deletion mutations in the LIYV CPm caused the loss of LIYV transmissibility by *Bemisia tabaci* biotype A (New World; Stewart et al., 2010), and elegant studies have recently demonstrated that the LIYV CPm specifically localizes and binds within the foregut of the vector whitefly, *Bemisia tabaci* biotype A (New World; Chen et al., 2011; and see Ng this volume). The studies of Stewart et al. (2010) also showed, however, that these same CPm deletions did not negatively affect systemic movement of the LIYV mutants in *N. benthamiana* plants. Interestingly, mutations in the three other virion proteins, CP, Hsp70h, and P59 resulted in the lack of the ability of these mutants to systemically infect *N. benthamiana* plants (Stewart et al., unpublished). These data suggest that LIYV virions lacking intact CPm can systemically invade *N. benthamiana* plants, but when any of the other three virion proteins are deleted, the ability to establish systemic infections was abolished.

## LIYV REPLICATION

The LIYV virion RNA analysis and nucleotide sequence data strongly suggested that LIYV had a bipartite genome. Still, all other closteroviruses known at that time were monopartite. Thus infectivity data for the LIYV RNAs were needed in order to address how these RNAs are replicated, and how their replication/gene expression was regulated. Predictive analyses based on nucleotide and deduced amino acid sequences suggested that RNA 1 encoded replication proteins while RNA 2 encoded “other” proteins. The development of a reverse genetics system for LIYV (Klaassen et al., 1996) enabled the opportunity to answer these and other fundamental questions. Initial studies were done using protoplasts prepared from a *N. tabacum* suspension cell culture (Passmore et al., 1993; Sanger et al., 1994). These were inoculated with LIYV virions and virion RNAs but only showed accumulation of positive and negative-sense LIYV RNA 1. The failure to obtain RNA 2 replication was surprising but showed that LIYV RNA 1 alone was replication competent (Klaassen et al., 1995). Since *N. tabacum* is not a systemic host for LIYV, additional studies further assessed the replication of LIYV RNAs 1 and 2 in mesophyll protoplasts that were prepared from *N. benthamiana* plants, a plant which is known to serve as a systemic host for LIYV. These studies showed that LIYV RNAs 1 and 2 were replication competent in these protoplasts. Therefore, full-length cDNA copies of LIYV RNAs 1 and 2 were developed, cloned into plasmids and used to generate *in vitro* transcripts that very closely resembled authentic LIYV RNAs 1 and 2. The RNA 1 and 2 transcripts, separately and in combination, were then used to inoculate *N. benthamiana* protoplasts. Northern blot analysis of extracts from protoplasts confirmed that LIYV RNA 1 alone was replication competent, and when co-inoculated with RNA 1, RNA 2 also accumulated in protoplasts. The pattern and intensity of hybridization signals were indistinguishable from those obtained from protoplasts that were inoculated with purified LIYV virion RNAs. Furthermore, progeny LIYV virions were observed in protoplasts inoculated with both RNAs 1 and 2 (Klaassen et al., 1996). These data suggested that the replication of LIYV RNA 2 was dependent on RNA 1. However, there are several distinct features, which suggest that LIYV replication may differ from other viruses in the genus *Closterovirus*.

The separation of LIYV replication and non-replication associated genes onto the two LIYV genomic RNAs suggests that this could offer a means to regulate replication and gene expression. Indeed, subsequent careful, time course analyses showed that the LIYV genomic RNAs show asynchronous temporal accumulation and gene expression when both RNAs are simultaneously inoculated to protoplasts. LIYV RNA 1 genomic and subgenomic RNAs accumulate to high levels almost 24 h before significant accumulation of RNA 2 can be detected (Yeh et al., 2000). This suggested that there is a fundamental difference in the replication of the two LIYV genomic RNAs; LIYV RNA 1 is likely to be replicated in *cis* while RNA 2 replication is in *trans* (Yeh et al., 2000).

These results also raised the further questions as to how LIYV RNAs 1 and 2 interact and presumably utilize the same replication complex within infected cells. Unlike most multipartite ssRNA plant viruses, the LIYV genomic RNA 1 and RNA 2 have very little nucleotide sequence homology within their 3′ terminal regions. This also is in contrast to what has been found for other criniviruses, which do show homology within their 3′ terminal regions. Could there be other proteins, besides ORF 1A and 1B that are required for LIYV RNA 2 replication and accumulation? Mutagenesis studies confirmed that LIYV RNA 2-encoded proteins do not affect RNA 1 and/or RNA 2 accumulation, but mutagenesis studies of the LIYV RNA 1 3′ end ORF encoding P34 gave an unexpected result. Although knockout mutations in this ORF did not affect the replication of LIYV RNA 1, they severely reduced the accumulation of LIYV RNA 2 (Yeh et al., 2000). These studies indicated that P34 is a *trans* enhancer of RNA 2 replication. Subsequent studies have shown additional properties for P34 further supporting a role in LIYV RNA 2 replication. Electrophoretic mobility shift competition assays using increasing concentrations of various unlabeled nucleic acids with fixed amounts of P34 and a ^32^P-labeled LIYV RNA 2 defective RNA [M5, the smallest replication-competent LIYV defective RNA 2 (Yeh et al., 2000)] showed that while ssRNA unlabeled competitors efficiently displaced the labeled M5 RNA, double-stranded RNAs (dsRNA), ssDNA, and dsDNA competitors did not, indicating that P34 is a ssRNA-binding protein. Furthermore, topology algorithms predicted that P34 is a membrane-associated protein, and deletion analysis mapped the P34 RNA-binding domain to its C-terminal region (Wang et al., 2010). One hypothesis is that perhaps P34 may be involved in targeting RNA 2 to LIYV replication sites within cells, or somehow protects or helps facilitate RNA 2 replication. Intracellular localization studies using a P34: green fluorescent protein (GFP) fusion showed that P34 exhibits perinuclear localization and that it colocalizes with the ER (Wang et al., 2010; **Figure 3**). The RNA 1 3′ ORF complement of other criniviruses is extremely variable (Dolja et al., 2006; Martelli et al., 2012a) and P34 shows no significant sequence identity even to proteins that are encoded by similarly positioned ORFs of other criniviruses. Whole plant studies with *Sweet potato chlorotic stunt virus* suggested a possible temporal accumulation of the SPCSV genomic RNAs 1 and 2 (Kreuze et al., 2002) similar to that seen for LIYV, but recent studies with *Lettuce chlorosis virus* (LCV) did not show obvious temporal regulation/accumulation differences among LCV RNAs 1 and 2 (Salem et al., 2009). Thus additional studies are needed to understand how other crinivirus RNAs 1 and 2 are replicated and interact within cells.

**FIGURE 5:**
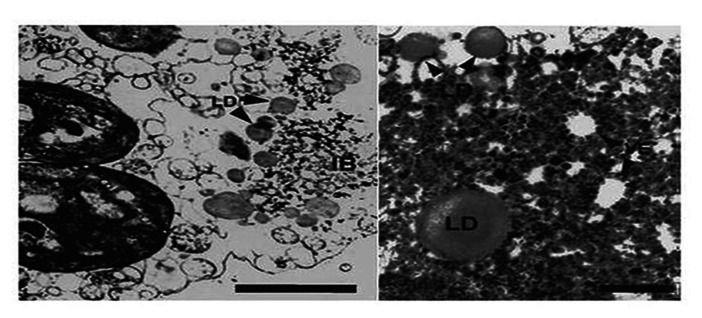
**Transmission electron microscopic (TEM) analysis shows lipid droplets (LD) that surround vesicles (VE) within the BYV-type inclusion bodies in LIYV RNA 1 and RNA2-infected *N. benthamiana* mesophyll protoplasts**. Image from Medina et al., 1998 with permission.

**FIGURE 6:**
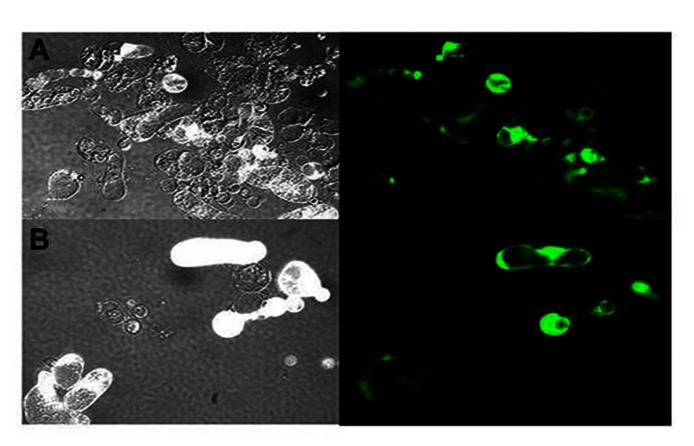
**Epifluorescent images on the morphology of protoplasts that are inoculated with transcripts for LIYV RNAs 1 and 2, and the M5 GFP defective RNA which is engineered to express the green fluorescent protein (GFP)**. Panel **(A)** shows control protoplasts (in bright field) inoculated with LIYV and M5 GFP and **(B)** shows fluorescence of protoplasts inoculated with LIYV and M5 GFP followed by cerulenin (50 uM) treatment. Cerulenin reduced LIYV infectivity (as assessed by protoplast fluorescence); however, in contrast, it did not have effect in TMV infectivity (see **Table 1**).

Although it has been realized for many years that viruses utilize host membranes as scaffolds for replication (den Boon et al., 2010; Laliberte and Sanfacon, 2010; Diaz and Ahlquist, 2012), how closteroviruses use host membranes has received relatively limited study. It has long been observed that closterovirus infections in plants result in extensive proliferation of ER membranes giving rise to characteristic vesiculated membranous inclusion bodies. This was originally observed for BYV giving rise to the name of “BYV-type inclusion bodies” (Lesemann, 1988). Abundant vesiculated membranous BYV-type inclusion bodies also can be found in plants (primarily within companion cells) and protoplasts infected by LIYV, and interestingly these also form in protoplasts infected by only LIYV RNA 1. LIYV-infected cells also show accumulations of lipid droplets surrounding these inclusion bodies (Medina et al., 1998; **Figure 5**), and LIYV infection induces re-organization of the ER (Wang et al., 2010). Together, these observations suggest that LIYV requires intact membranes for RNA replication. This is further supported by our recent preliminary studies where the effects of two drugs, cerulenin and brefeldin A, were used to assess LIYV replication levels in *N. benthamiana *protoplasts (unpublished). Cerulenin inhibits *de novo* fatty acid and steroid biosynthesis. Cerulenin binds in equimolar ratios to β-keto-acyl-ACP synthase, thus blocking its interaction with malonyl-CoA thereby affecting fatty acid synthesis. In contrast, brefeldin A inhibits the transport of proteins from the ER to Golgi, and also induces retrograde protein transport from the Golgi to the ER (Klausner et al., 1992). We used fluorescence microscopy and northern hybridization analysis and found that while cerulenin greatly reduced LIYV infection and accumulation in protoplasts, we saw no detectable effects on TMV (*Tobacco mosaic virus*; **Table 1** and **Figure 6**-unpublished). In contrast, brefeldin A, although resulting in earlier cell death, showed no effects on LIYV, but showed a slight increase in TMV replication in protoplasts as measured by GFP fluorescence. The observations give additional support that LIYV replication depends on both ER-derived membrane recruitment and *de novo* biosynthesis of lipids.

**Table 1:** Effects of cerulenin and brefeldin A on LIYV and TMV infectivity in *N. benthamiana* protoplasts.

	LIYV^1^	TMV^2^
Control	21.5 ± 8.7^3^	39.6 ± 1.3
Cerulenin 50 μM	0.43 ± 0.3	33.3 ± 9.6
Brefeldin A 10 μg	20.6 ± 8.7	53.5 ± 21.9

1 Cells were inoculated with transcripts for LIYV RNAs 1 and 2, plus the M5-GFP.

2 Cells were inoculated with transcripts for TMV GFP 30B.

3 Percentage of GFP fluorescent cells.

## NON-VIRION PROTEINS

**FIGURE 7:**
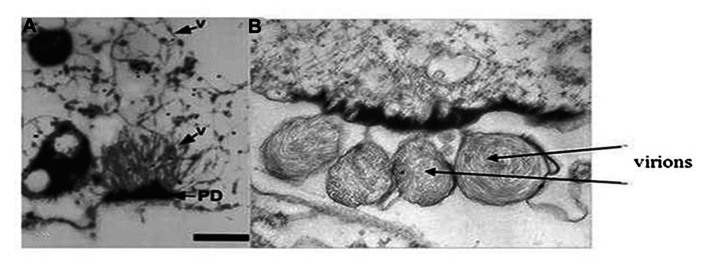
**Transmission electron micrographs showing (A) virions (see arrow and V) extending from the plasmalemma deposits (PD) and into the cytoplasm of a LIYV-infected *N. benthamiana* protoplast (image is from Medina et al., 1998 with permission)**. Panel **(B)** shows sacks of LIYV virus-like particles external to the plasmalemma, directly adjacent to abundant plasmalemma deposits in a single *N. benthamiana* protoplast.

While much recent research has focused on studying the genomic RNA sequences, genome organization, and phylogenetic relationships for many newly discovered criniviruses, there are still many crinivirus-encoded proteins whose roles in infections have not been elucidated. For example, LIYV RNA 2 encodes for a protein at its 3′ terminus, P26. Similarly positioned genes encoding similarly sized proteins are found among all criniviruses sequenced to date. LIYV P26 is not a virion component (Tian et al., 1999) and microscopic studies have shown that P26 associates with, unique to LIYV, plasmalemma deposits (PLDs) (Medina et al., 2005; **Figures 7A,B**). Plasmalemma deposits were first described by Pinto et al. (1988) and Hoefert et al. (1988) in their extensive and beautiful electron microscopic studies of LIYV infection development in lettuce plants. They showed that the plasmalemma deposits were primarily found in companion cells, often in pit fields and adjacent to plasmodesmata connections to sieve elements. They noted that the plasmalemma deposits frequently had what appeared to be LIYV virus particles associated with them and these were oriented perpendicular to the plasmalemma. They also observed what appeared to be LIYV virions in adjacent sieve tube cells exiting plasmodesmata (**Figure 1F**) and speculated that the plasmalemma deposits might have roles in transporting LIYV virus particles from companion to sieve tube cells. Recent studies have shown that the plasmalemma deposits also are formed in LIYV-infected protoplasts, and they also contain aggregates of LIYV virions arranged perpendicular to the plasmalemma. “Sacks” of LIYV virions can also be found external to the plasmalemma of LIYV-infected protoplasts immediately adjacent to the plasmalemma deposits (**Figures 7A,B**). Moreover, further studies showed that P26 still associates to plasmalemma deposits when expressed from the heterologous TMV vector indicating that no other crinivirus protein is needed for this association. Hence, P26 association to the plasmalemma deposits is unique (Stewart et al., 2009b). Interestingly, interaction studies indicated that P26 is also capable of self-interaction (Stewart et al., 2009a). It is possible that P26 might be interacting with LIYV virion components due to its association with PLDs, or with other host factors to facilitate the movement of LIYV virions either within cells directing them to the cell periphery and/or through the plasmodesmata that connects the cells. To elucidate the functions of P26 has been quite challenging since it has shown to be non-essential for LIYV replication processes in protoplasts (Yeh et al., 2000). Whole plant infections of LIYV mutants derived from protoplasts has been difficult to achieve (Ng and Falk, 2006). More recent whole plant inoculations using *Agrobacterium tumefaciens* to deliver specific LIYV mutants to *N. benthamiana* plants (Grimsley et al., 1986; Wang et al., 2009) have shown in a few experiments so far that P26 mutants do not systemically infect *N. benthamiana* plants (unpublished), further supporting a role for LIYV P26 in systemic plant infection.

It is worthwhile to note that LIYV RNA2 encodes two other proteins. ORF 1 encodes a small hydrophobic protein, P5, with a transmembrane helix (Klaassen et al., 1995). The orthologous gene (protein) in BYV has been shown to play an important role in the cell-to-cell movement of BYV, and it has been indicated to be localized to the ER as a type III integral membrane protein (Alzhanova et al., 2001; Peremyslov et al., 2004; **Figure 2**).

Another small protein, P9, is also encoded on LIYV RNA 2. Although no functions have yet been assigned to this protein, a P9-like protein is predicted to be encoded by a similarly positioned ORF in all of the members of the genus *Crinivirus* sequenced so far (**Figure 2**). However, this protein shows high sequence variability among these viruses. Yeast two hybrid studies of P5 and P9 with each other and with the other LIYV RNA 2-encoded proteins showed that P9 is self-interacting (Stewart et al., 2009a). Whether these P9 protein homologs in other criniviruses also show self-interaction remains to be determined. Further studies are underway to determine roles of P9, and/or P5 in LIYV infections. Reverse-genetics systems have currently been established to elucidate the possible functions of these proteins in both *in planta* as well as via protoplast inoculation to investigate possible roles in replication. The presence of ORFs encoding these proteins in all crinivirus genomes sequenced so far indicate important roles and possible interactions with host factors, and thus, playing important roles in the virus life cycle.

## CONCLUSIONS AND PERSPECTIVES

We have attempted to give an overall picture of what is known, and some things that remain to be studied for an understanding of crinivirus replication and host interactions. Although more and more criniviruses are being identified and their genomes are being sequenced, we are in need of more fundamental studies on their biology and molecular biology. LIYV has served well as a model crinivirus, but it is interesting to note that phylogenetically, LIYV is not closely related with the majority of the criniviruses. Studies with other criniviruses might give different information, or validate LIYV as a good model crinivirus.

Presently, excellent progress is being made in gaining a better understanding of crinivirus:whitefly interactions (Stewart et al., 2010; Chen et al., 2011; and see Ng this volume). This is important, and no doubt will lead to new fundamental information on the complex biology of criniviruses, but also could lead to novel strategies for controlling diseases caused by various criniviruses. By contrast, we still know too little about crinivirus:host plant interactions, and in particular how criniviruses move within the phloem. We have alluded to some ideas for LIYV *in planta* movement such as possible roles of plasmalemma deposits of LIYV P26, and these are based on biological studies as well as excellent electron and more recently light microscopic analyses of plants and even protoplasts (Hoefert et al., 1988; Pinto et al., 1988; Medina et al., 2005; Stewart et al., 2009b). Novel and important recent studies with GFP-tagged CTV have given new insights into how CTV multiplies and spreads within phloem cells of different citrus types (Folimonova et al., 2010), and such accomplishments with criniviruses are needed.

Also needed is a greater understanding of crinivirus replication and host interactions. Clearly, the temporal regulation seen for LIYV is intriguing, and all criniviruses have the same dilemma when virions are inoculated into a cell: how do the two genomic RNAs get together at the same intracellular location so both RNAs can express their genetic information and be replicated, ultimately to yield progeny virions? The identification almost 50 years ago of the membranous vesiculated “BYV-type inclusion bodies” (Lesemann, 1988) suggests a role for cellular membranes in at least BYV replication, and our studies with LIYV suggest this as well. Our recent demonstration of the negative effects of cerulenin on LIYV replication further supports the role of the endomembrane system for supporting LIYV replication. These studies could potentially lead to the development of novel control strategies against criniviruses that are current threats in world agriculture.

Despite their economic importance and widespread incidence in various host plants almost worldwide, reverse genetics systems are now available for only two criniviruses: LIYV and LCV (Wang et al., 2009; Chen et al., 2012). Both can be delivered to at least *N. benthamiana* plants via agroinoculation, but it can still be a challenge to efficiently deliver these phloem limited viruses to plants, particularly to hosts other than *N. benthamiana*, and improvements here would be very important. Current studies, particularly in herbaceous plants, require the use of the appropriate crinivirus whitefly vector for virus studies. This can be difficult to impossible in some locations (e.g., if the virus or whitefly vector is an exotic pathogen/pest) and hinders even opportunities to rapidly screen germplasm for virus resistance. Effective delivery to natural hosts of specific criniviruses by agroinoculation, or some other means not relying on the whitefly vector would yield great practical as well as fundamental benefits.

Although many criniviruses presently cause important diseases in many crop plants, successful strategies for their control are limited. In areas where vector populations are high, insecticides are often used but generally are ineffective in preventing crinivirus inoculation to susceptible plant hosts. It is interesting that no successful genetically engineered approaches capable of inducing RNA interference-based immunity are known for criniviruses. This is despite efforts at least with SPCSV in sweet potatoes (Kreuze et al., 2008). However; there have been some improvements with other members of the family *Closteroviridae*, such with CTV (Soler et al., 2012). Genetically engineered resistance against many different plant viruses will be a part of future disease control strategies, and a greater understanding of crinivirus replication and host plant interactions might allow for opportunities to effectively use such strategies to control diseases caused by criniviruses.

## Conflict of Interest Statement

The authors declare that the research was conducted in the absence of any commercial or financial relationships that could be construed as a potential conflict of interest.
